# Deciphering the genetic diversity and population structure of wild barley germplasm against corn leaf aphid, *Rhopalosiphum maidis* (Fitch)

**DOI:** 10.1038/s41598-023-42717-7

**Published:** 2023-10-12

**Authors:** Sunny Maanju, Poonam Jasrotia, Surender Singh Yadav, Prem Lal Kashyap, Sudheer Kumar, Manoj Kumar Jat, Chuni Lal, Preeti Sharma, Gyanendra Singh, Gyanendra Pratap Singh

**Affiliations:** 1 ICAR-Indian Institute of Wheat and Barley Research, Karnal, Haryana 132001 India; 2 CCS Haryana Agricultural University, Hisar, Haryana 125004 India; 3 ICAR-National Bureau of Plant Genetic Resources, New Delhi, 110012 India

**Keywords:** Biotechnology, Molecular biology

## Abstract

Corn-leaf aphid (CLA-*Rhopalosiphum maidis*) is a major insect pest of barley (*Hordeum vulgare*) causing yield loss upto 30% under severe infestation. Keeping in view of the availability of very few sources of CLA resistance in barley, the present investigation was framed to assess the genetic diversity and population structure of 43 wild barley (*H. vulgare* subsp. *spontaneum*) genotypes using eight microsatellite markers against *R. maidis*. Three statistical methods viz*.* multivariate-hierarchical clustering, Bayesian clustering and PCoA, unanimously grouped genotypes into three subpopulations (K = 3) with 25.58% (SubPop1-Red), 39.53% (SubPop2-Green) and 34.88% (SubPop3-Blue) genotypes including admixtures. Based on Q ≥ 66.66%, 37.20% genotypes formed a superficial “Mixed/Admixture” subpopulation. All polymorphic SSR markers generated 36 alleles, averaging to 4.5 alleles/locus (2–7 range). The PIC and H were highest in MS31 and lowest in MS28, with averages of 0.66 and 0.71. MAF and mean genetic diversity were 0.16 and 89.28%, respectively. All these parameters indicated the presence of predominant genetic diversity and population structure amongst the studied genotypes. Based on AII, only 6 genotypes were found to be *R. maidis* resistant. SubPop3 had 91.66% (11) of the resistant or moderately resistant genotypes. SubPop3 also had the most pure genotypes (11), the least aphid infestation (8.78), and the highest GS (0.88), indicating its suitability for future *R. maidis* resistance breeding initiatives.

## Introduction

Barley (*Hordeum vulgare* L.) is the fourth most low-input cereal crop after maize, wheat, and rice^1^, and is currently grown on 52.66 mha of land worldwide^2^. It is used for food, feed and for malt purpose^3^. Due to its hardiness, it is an excellent choice for growing on marginal lands as it is highly tolerant to drought, heat, cold and salinity^1^. Cultivated and wild barley grow in a wide range of natural and man-made habitats^4^. Historically, the cultivated barley (*H. vulgare* subsp. *vulgare*) was domesticated in the F.C. (Fertile Crescent of West Asia) area about 8000–12,000 years ago from its progenitor, *H. vulgare* subsp. *spontaneum* (wild barley). The only region where wild and cultivated barley are still growing together is F.C. (modern-day Israel, Syria, Iraq, Lebanon, Jordan and Palestine, together with parts of northern Kuwait, southeastern Turkey and western Iran)^5–10^. It has been observed that development of elite barley cultivars and their domestication had resulted in reduction of number of alleles^11^ and showed reduced genetic diversity as compared to the wild genotypes^12–14^. The small genetic basis of cultivated barley has been a crucial gap in the breeding process therefore, the wide genetic variability of wild barley may provide more number of alleles for breeding and development of superior barley cultivars^15,16^. Furthermore, in comparison to the genetic similarity of modern crop varieties, wild genotypes exhibit variation both within and between populations which may aid them to overcome environmental stresses^17^. Understanding the type and degree of population structure and genetic diversity in wild barley genotypes is critical for effective maintenance and deployment of promising trait specific germplasm for the development of insect resistance cultivars.

Globally, a total of seven aphid species have been reported to attack barley crop by Weibull et al. ^18^. Amongst, *R. maidis* belonging the order Hemiptera and family Aphididae is the most serious insect pest of barley and it is also reported as the most preferred host of the CLA^19^ causing around 17.1–30% yield losses^20–22^. Development of insect-resistant cultivars is an environment friendly, effective and easy to use method for the farmers^23^ over use of environmentally hazardous insecticides. However, none of the presently sown barley cultivars is resistant to *R. maidis*^24,25^. The resistance to *R. maidis* is found to be controlled by one or two recessive or dominant genes^25–29^. Two markers SCSSR15864 and KV1/KV2 were found to be effective in identifying CLA resistance barley genomic region^30^. Identification of underlying resistance mechanisms as well as morphological and biochemical characterisation of aphid resistance in barley are currently desperately needed. It has been well documented that wild barley, although more of a self-pollinated type, still has wide genetic diversity^31^. Resistance genes from wild species can be translocated into cultivated variety by introgression breeding. Ethiopian barley cultivars have shown substantial diversity in resistance to barley shoot fly^32^ and major disease resistance^32,33^.

For the conservation of the genetic diversity as well as appropriate use of this accessible diversity in breeding, systemic molecular assessment using molecular markers is a must. Evaluation of genetic diversity has been carried out using simple sequence repeats (SSRs) markers in several studies. Systemic molecular evaluation utilising molecular markers is required for the conservation of genetic diversity as well as the optimal utilisation of this accessible diversity in breeding. In various studies, genetic diversity has been assessed using simple sequence repeats (SSRs) markers. It was reported that the most diversity parameters can lead to significant variations in barley germplasm^34–37^. Although, many of these studies used either cultivars^38,39^, or collections of landraces and cultivars^40–42^ or only landraces^43^ from different geographical locations. Furthermore, wild barley germplasm have been studied using the advancement in genetic marker technologies like AFLPs^44^, RAPDs^45^, isozyme^46^, SNPs^47^, DarT array^48^ and SSRs^49^. When compared to other types of DNA markers, PCR-based microsatellite (SSR) markers have been widely used for genetic diversity and population structure analysis in barley germplasm due to their ease of use, high polymorphism, reproducibility, cost-effectiveness, abundance in genome, codominance, and multiallelic nature^50–58^. In order to analyse diversity amongst genotypes, conserve resources, and utilise noble alleles, more effective molecular markers would be beneficial^59^.

The species crossover system, natural selection, genetic drift, and gene flow all have a substantial effect on the genetic make-up of plant populations including sudden modifications in the climate^60^. For targeting higher yield, quality disease and pest resistance, it is crucial to have a complete understanding of genetic variations across and within populations as well as their genetic relatedness among germplasm resources^61^. Many a times the information on genetic diversity between and within barley genotypes are not known and even if they are known, then the knowledge on the pattern and level of this diversity within genotypes is not well researched^62^. The geographical proximity of genotypes does not necessarily represent the genetic structure of populations. Sometimes, due to unknown impediments to gene flow, populations which are not spatially dispersed can still be structured genetically. Moreover, groups of genotypes with various phenotypes or behavioural traits from different geographical regions are not always genetically distinct from one another^63,64^. Keeping in view, it is pertinent to assess the genetic variation and population structure parameters to find novel sources of genetic variation with tolerant response to insect damage which can be utilized in breeding programmes. *Hordeum* sp. apart from cultivated barley are seen as potential *R. maidis* resistance sources. *H. bogdani,* a diploid species, had high resistance levels, but the transfer of resistance to cultivated barley has been unsuccessful due to genetic incompatibilities between *H. vulgare* and this species^24^. The genetic associations analysis between crops is a vital component for crop improvement because it forms the basis for selection of parental lines for hybridization, analyse genetic variability of genotypes and identify germplasm that need to be preserved to protect highest genetic variability. In light of the foregoing, and in order to fill research gaps, the current study was planned to study the genetic diversity and population structure analysis in barley to identify sources of resistance against the corn-leaf aphid, *R. maidis*, using microsatellite markers.

## Results

### Population stratification

**Figure 1:**
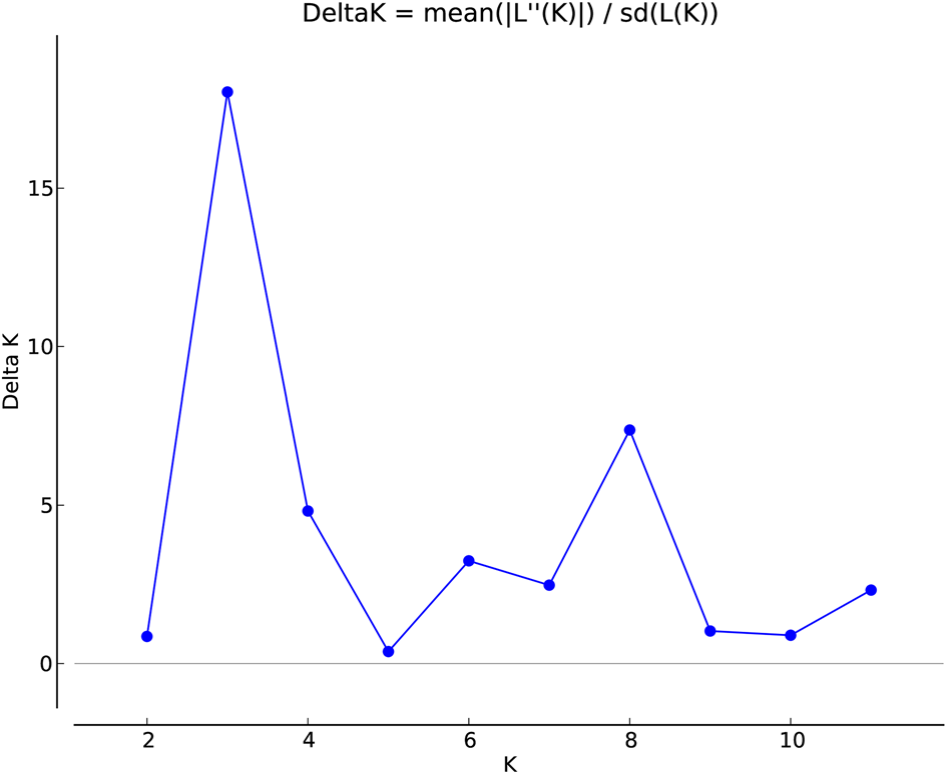
Estimation of the genetically most probable number of *H. vulgare* subsp*. spontaneum* subpopulations based on Evanno’s Delta K (ΔK) method. The maximum value of delta K occurred at K = 3 indicates that the investigated wild barley genotypes can be divided into three hypothetical subpopulations.

On the basis of raw data of the electrophoretic bands generated from markers, the STRUCTURE analysis for K = 1 to K = 12 revealed the most probable subpopulation number at K = 3 (Fig. 1). It was found after running the data through the online STRUCTURE Harvester website by following the ΔK method and plotting the graph between LnP(D) log likelihood data values and assumed successive K values.

**Figure 2:**
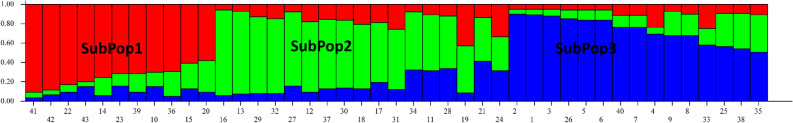
Bar plots for 43 wild barley genotypes generated by STRUCTURE using the admixture model and sorted by Q at K = 3, with independent allele frequency based on binary data of 8 SSR loci. The groups (subpopulations) are represented by different colors. Each bar represents one barley genotype (identified by genotype code) and is partitioned into segments indicating its genetic composition similarity with other genotype groups.

The 43 wild barley (*H. vulgare* subsp*. spontaneum*) genotypes were grouped into three clusters (as K = 3) of different colours viz. SubPop1 (Red), SubPop2 (Green) and SubPop3 (Blue) with 9, 7 and 11 genotypes, respectively that purely belonged to these subpopulations (Table 1) and sorted by cluster membership coefficient (Q) (Fig. 2) at 0.6666 (66.66%). Due to heterogeneous allelic trends in sixteen genotypes, none of the subpopulations could be purely ascribed to them by the STRUCTURE software and hence they were designated as Mixed subpopulation. However, these admixture genotypes categorized as superficial Mixed subpopulation were allotted to one of the original subpopulations which had highest Q value among all within a genotype (Table 1). So, the admixtures of different subpopulation were: 2 (genotype code: 15 and 20) for SubPop1, 10 (genotype code: 11, 17, 18, 19, 21, 24, 27, 28, 31 and 34) for SubPop2 and 4 (genotype code: 25, 33, 35 and 38) for SubPop3. Overall, the three subpopulations (SubPop1, SubPop2 and SubPop3) including their admixture genotypes represented 25.58%, 39.53% and 34.88% genotypes of the total population size, respectively.

**Table 1:** Proportion of 43 wild barley genotypes based on Cluster membership probability coefficient (Q) to different subpopulations derived from STRUCTURE software.

Subpopulations	Pure genotypes (Q ≥ 66.66%)	Admixture genotypes (Q ≤ 66.66%)	Total
SubPop1 (Red)	9 (20.93%)	2 (4.65%)	11 (25.58%)
SubPop2 (Green)	7 (16.29%)	10 (23.25%)	17 (39.54%)
SubPop3 (Blue)	11 (25.58%)	4 (9.30%)	15 (34.88%)
Total	27 (62.80%)	16 (37.20%)	43 (100%)

Based on the percentages of membership (Q) of genotypes in each of the three clusters, it was found that twenty-seven genotypes, which correspond to 62.79% of the total genotypes, had a Q value higher than 0.6666 (or 66.66%). This indicated that these genotypes are predominantly assigned to one of the three clusters with a relatively high degree of certainty. Seventeen genotypes, accounting for 39.53% of the total genotypes, had a Q value higher than 0.75. These genotypes showed an even stronger membership in their assigned clusters compared to the previous category. Eight genotypes, representing 18.60% of the total genotypes, had a Q value higher than 0.85. These genotypes demonstrate a high level of membership in their respective clusters, suggesting a clear assignment to a specific subpopulation. Only two genotypes, making up 4.65% of the total genotypes, had a Q value higher than 0.90. These genotypes exhibit an exceptionally strong membership in their assigned clusters, indicating a very high degree of certainty in their classification. Overall, the results suggest that the majority of genotypes have reasonably high level of membership probabilities in their respective clusters.

The comparison of genotypes grouped in different subpopulations to their places of origin revealed that all the subpopulations had genotypes from all five places of origin (Israel, Jordan, Libya, Syria and Tajikistan) except that SubPop1 was not present in Israel (Supplementary Table S1). This indicates the high degree of variation in the grouped subpopulations. An admixture genotype IG 144898 (genotype code 21) was the only genotype from the sixth place of origin, the Palestinian Territory (Supplementary Table S1). There was no specific pattern between the places of origin and the resistance reaction of the genotypes.

### Aphid resistance diversity pattern

**Figure 3:**
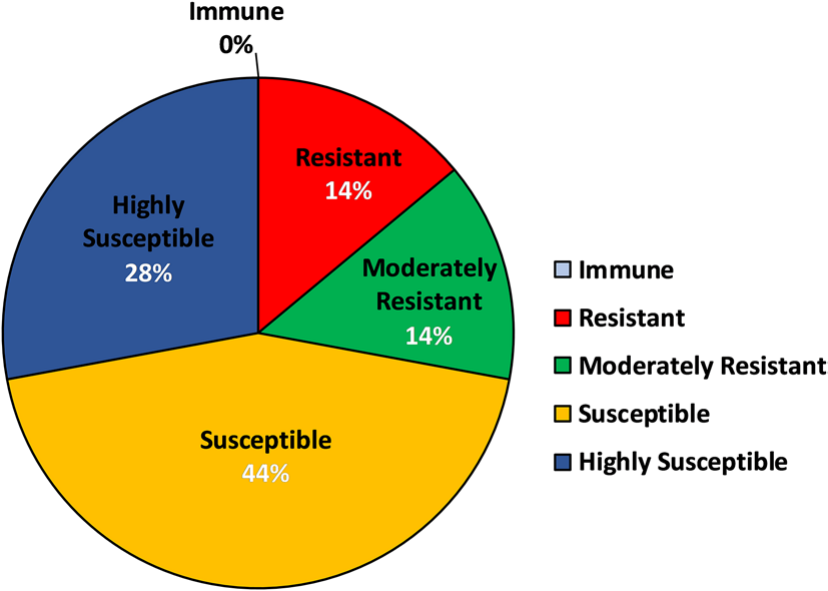
Pie Chart depicting characterization of wild barley genotypes into categories of resistance based on AII (Aphid Infestation Index). The colours in the pie-chart are not used as a references for indicating subpopulations.

In order to determine the aphid preference to the various barley genotypes used in the present investigation, an Aphid Infestation Index (AII) was devised on weightage basis, of which highest was given to the number of aphids infesting per shoot, followed by the sequence of symptoms of aphid damage like leaf chlorosis and leaf rolling. Based on the AII, all the genotypes were characterized into 5 categories of resistance response viz. immune, resistant, moderately resistant, susceptible and highly susceptible (Supplementary Table S3 online) with 0%, 14%, 14%, 44% and 28% genotypes (Fig. 3), respectively. There was no immune genotype identified in the current study, however, a total of 12 genotypes, which represented 28% of the total barley genotypes were either found to be moderately resistant or resistant (Fig. 3). IG 135854 (genotype code 5), IG 142356 (genotype code 6), IG 142486 (genotype code 7), IG 144112 (genotype code 8), IG 144113 (genotype code 9) and IG 144114 (genotype code 10) were the only six genotypes which emerged as resistant to corn-leaf aphid, *R. maidis* (Supplementary Table S3 online)*.*

**Figure 4:**
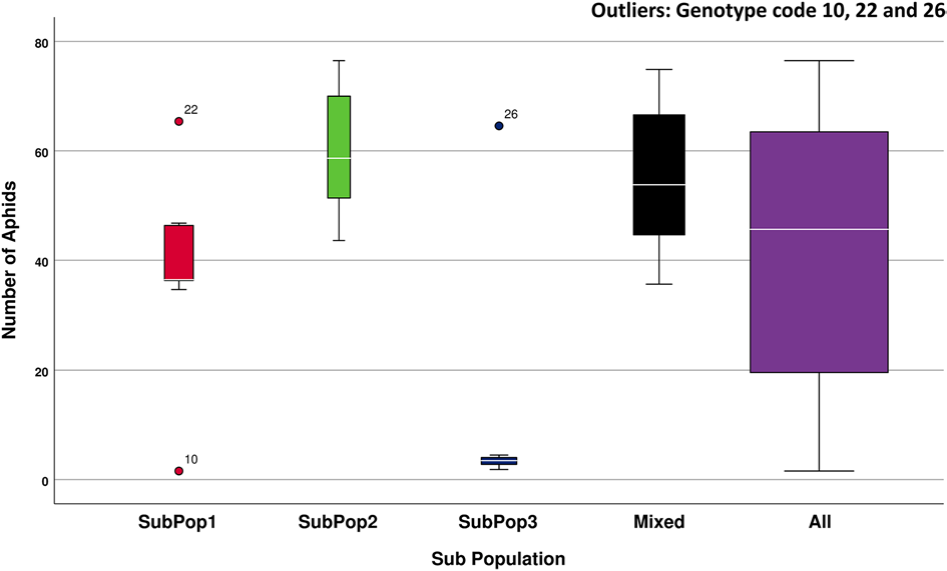
Descriptive statistics of number of *R. maidis* aphids infecting all 43 wild barley genotypes and when divided into three subpopulations derived from cluster analysis.

Descriptive statistics is given in the form of box plot diagram indicating number of aphids infesting per shoot coloured and divided into subpopulations as revealed by cluster analysis, have been shown in the Fig. 4. The width of the box is representative of the number of genotypes included in that subpopulation. The ANOVA results suggested that the mean number of aphids infesting genotypes of various subpopulations differed significantly (*F*_4,81_ = 9.848, *p* < 0.05). The equal variance assumption was not made since the Levene's statistic was significant (*p* < 0.05). The Dunnett's T3 test was used to evaluate post-hoc comparisons in order to look for individual differences amongst subpopulations. The test indicated that the mean number of aphids infesting SubPop3 (M = 8.78, SD = 18.51) was significantly (*p* < 0.05) distinct from the mean number of aphids infesting SubPop1 (M = 38.63, SD = 16.85) and SubPop2 (M = 60.21, SD = 12.81). Mean number of aphids infesting SubPop3 (M = 8.78, SD = 18.51) also differed significantly (*p* < 0.05) from the superficial Mixed subpopulation (M = 55.04, SD = 12.63) and the mean number of aphids infesting all of the subpopulation combined together (M = 40.61, SD = 25.01). The mean differences among subpopulations mentioned above were significant at the 5% level of significance. However, no significant differences were observed between other subpopulation combinations. IG 144114 (genotype code 10) and IG 144903 (genotype code 22) were the outliers of SubPop1, while IG 144930 (genotype code 26) was the outlier of SubPop3. Since, SubPop3 (Blue) had the highest number of pure genotypes (i.e., 11) in it along with the least mean number of aphids (8.78) infesting its genotypes, it emerged as the most favourable group for search of aphid resistance.

**Figure 5:**
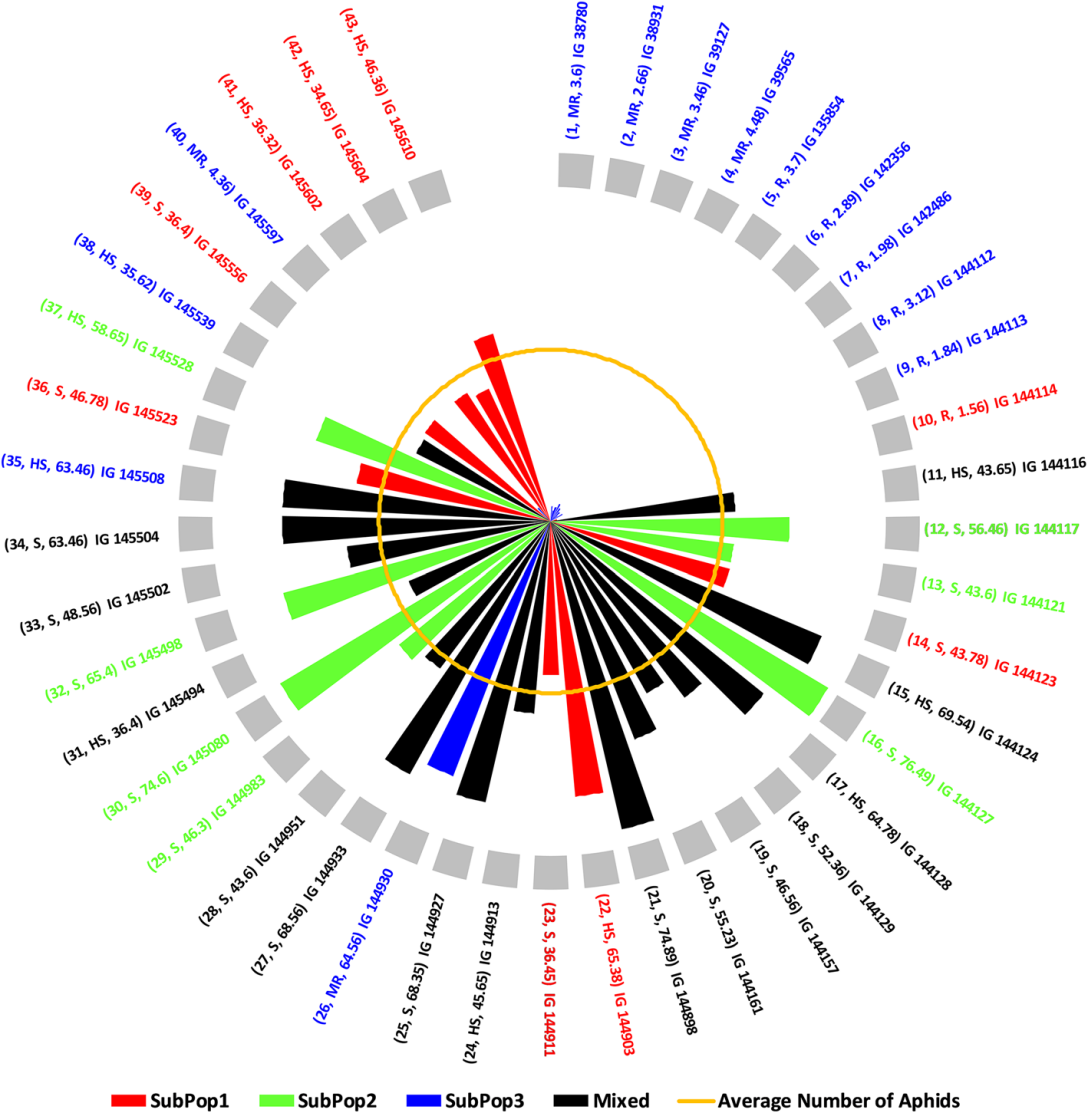
Wind-Rose Chart based on mean number of aphids infesting 43 wild barley genotypes depicting resistance reaction (AII) and subpopulations (STRUCTURE). Each line representing a genotype has been coloured according to the colour of subpopulation to which the genotype belongs as revealed by STRUCTURE. The inner yellow circle represents the average number of aphids on all the genotypes. Each genotype has been labelled in the order: (Genotype code, Resistance reaction based on AII, Mean number of aphids infesting the genotype) Name of the genotype.

Overall, Fig. 5 combines the information on mean number of aphids infesting per shoot, resistance reactions of genotypes based on AII and the subpopulations divided by STRUCTURE cluster analysis, in the form of a wind-rose chart. On comparison, 91.66% (11 in total) of the genotypes belonging to either resistant or moderately resistant category were found to be from SubPop3 (Blue). Five of the six resistant genotypes, identified based on AII, IG 135854 (genotype code 5), IG 142356 (genotype code 6), IG 142486 (genotype code 7), IG 144112 (genotype code 8) and IG 144113 (genotype code 9) also belonged to the SubPop3.

### Analysis of genetic diversity

The germplasm of 43 wild barley varieties was amplified using eight microsatellite markers, with their primer sequences and repeat motifs are given in Table 2. The markers produced a total of 36 polymorphic bands, with an average of 4.5 bands per marker and a range of 2–7. The PIC, which ranges from 0 to 1, is a measure of the degree of polymorphism, which in this study varied from 0.35 (MS28) to 0.80 (MS31) for the corresponding SSR loci. The mean PIC value of all eight markers investigated in this study was 0.66 (Table 2), indicating that they are highly polymorphic and informative (PIC > 0.50). Seven out of eight markers (87.5%) were highly informative (PIC > 0.50), while only MS28 (PIC = 0.35) was medium informative marker (0.5 > PIC > 0.25). While, Heterozygosity (H) which indicates the average frequency of heterozygous individual occurrence was found to be highest in MS31 (0.83) and lowest in MS28 (0.44), with 0.71 average H. The genetic diversity parameter averaging to 0.16 for the tested markers ranged between 0.02 and 0.27. MAF varied from 76.74 to 98.84%. On an average at any locus, 89.28% of the 43 wild barley genotypes had a common major allele. The number of bands observed in subpopulations ranged from 12 (SubPop2 and SubPop3) to 26 in SubPop1, which all had a frequency of more than 5%. This study reported 18 unique or private alleles from 19 wild barley genotypes spread across each and every subpopulation.

**Table 2:** Genetic characteristics of 8 polymorphic SSR loci in 43 wild barley genotypes. *F* forward primer, *R* Reverse primer, *PIC* Polymorphic Information Content, *H* Heterozygosity.

Locus	Primer sequence (5′–3′)	Repeat motif	Amplicon size (bp)	Number of alleles	Major allele frequency (%)	Gene diversity	PIC	H
MS3	F: TAGCCTTTGAGGTCGATGTAGG	(GTT)_5_	150–350	5	86.98	0.21	0.75	0.78
	R: TGGGTGTCTTTCAGATGAGTTG							
MS17	F: TCTTGTGGAGTCTGCTGTTGTT	(TGC)_5_	300–600	4	94.19	0.10	0.69	0.73
	R: GTAGCTTCAGGTCGCATCACTT							
MS20	F: GGTTGTTGTCATAGGGGTTGTC	(TCG)_4_	100–500	6	92.64	0.12	0.76	0.79
	R: TACCAGAACATGGGTTTCAGC							
MS21	F: AGGTCTGGTGTGAGTGTTGATG	(TGA)_4_	200–300	3	93.80	0.11	0.58	0.66
	R: CTCCTCATTGTAGTGCGTGTGT							
MS25	F: TACTTCTCCTCCTCCTCCTCCT	(TATT)_5_	150–700	5	81.40	0.26	0.71	0.75
	R: GAACTCGCAAAGTGGTTTCTCT							
MS27	F: CATTTCAGTGTTGGACAAGCAT	(GTGTCA)_4_	200–600	4	76.74	0.27	0.66	0.71
	R: AGAGAGTTTCGTAGTTGGGCAG							
MS28	F: CTAAGCATAAGGAGGCAACCAG	(TAAAA)_5_	150–300	2	98.84	0.02	0.35	0.44
	R: CGGAGTATTGGGAGTGAAATGT							
MS31	F: CACAAACACACACACACACACA	(GCTCCC)_4_	150–800	7	89.70	0.18	0.80	0.83
	R: CTGAACAGTAAAGCCTGAAGGG							
Average	–	–	–	4.5	89.28	0.16	0.66	0.71

**Figure 6:**
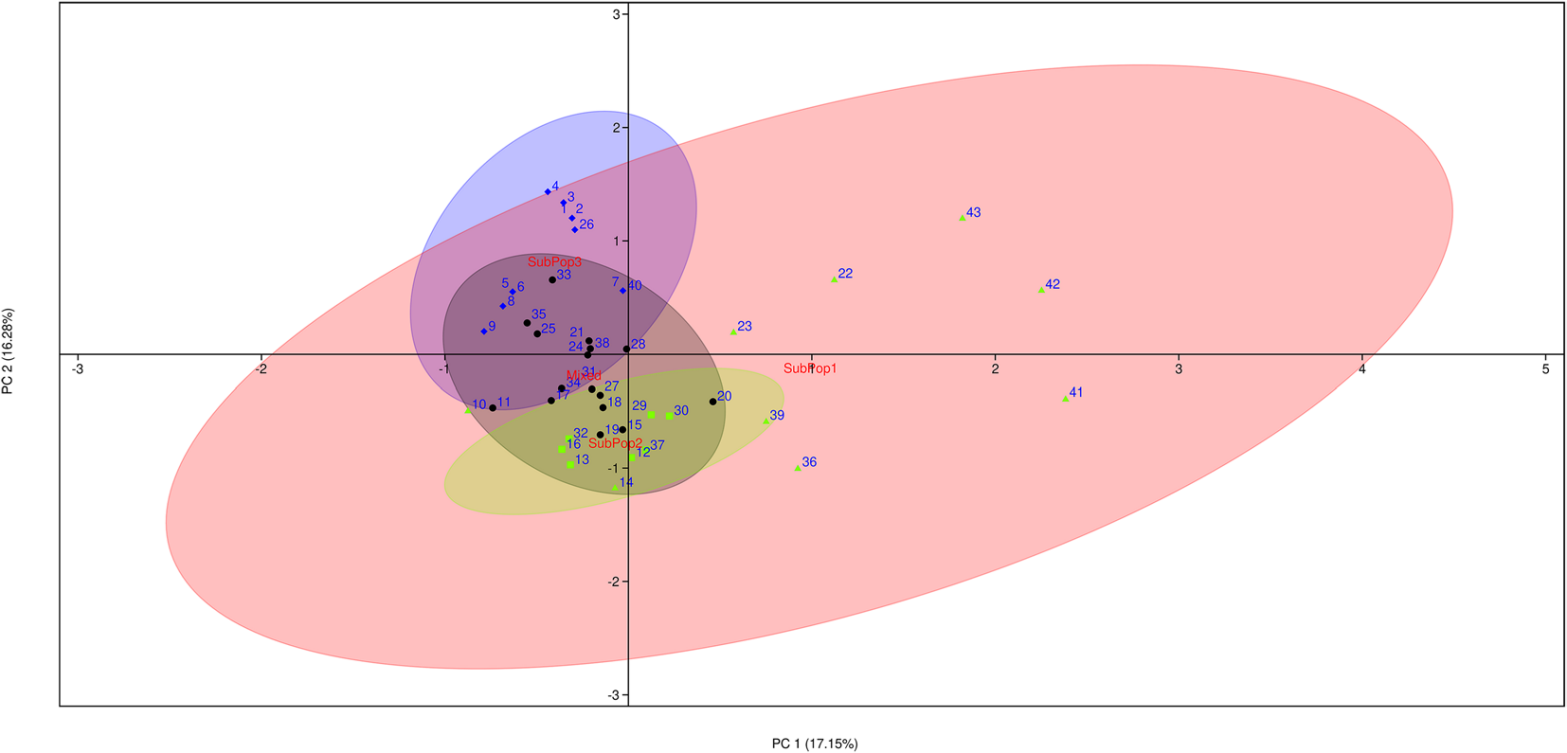
Principal coordinates analysis (PCoA) on 43 wild barley genotypes based on 8 SSR markers. Different colours indicate different subpopulations (SubPop1–SubPop3) in the population as revealed by STRUCTURE software.

The genetic similarity between and within subpopulations as well as within whole of the population was assessed using Jaccard's coefficient in order to further define the genetic relationship between the different barley genotypes. Based on NTSYS-pc output calculation, the 43 wild genotypes of barley had an average genetic similarity of 0.82 and a range of 0.55–1.00, demonstrating the genotypes' considerable genetic heterogeneity. The GS of paired barley genotypes in each subpopulation revealed that SubPop1 (Red) had the largest range (0.55–0.91), showing the greatest genetic diversity among all, and the lowest GS coefficient (0.74). SubPop3 was found to have the greatest genetic similarity coefficient, 0.88. Principal coordinate analysis (PCoA) was used as an alternative approach for evaluating and visualising population structure. The first three principal coordinates explained 44.51% of the genotypic variation (PC1: 17.15%, PC2: 16.28%, PC3: 11.08%) and distinguished SubPop1, SubPop2, SubPop3, and SubPop4 genotypes with 95% confidence ellipses, corroborating the STRUCTURE finding (Fig. 6). These subpopulations have been coloured in accordance with the colours generated by STRUCTURE software for easy comparison. The intersection of the different subpopulation clusters indicates the interaction among them and high genetic diversity among the subpopulations.

**Figure 7:**
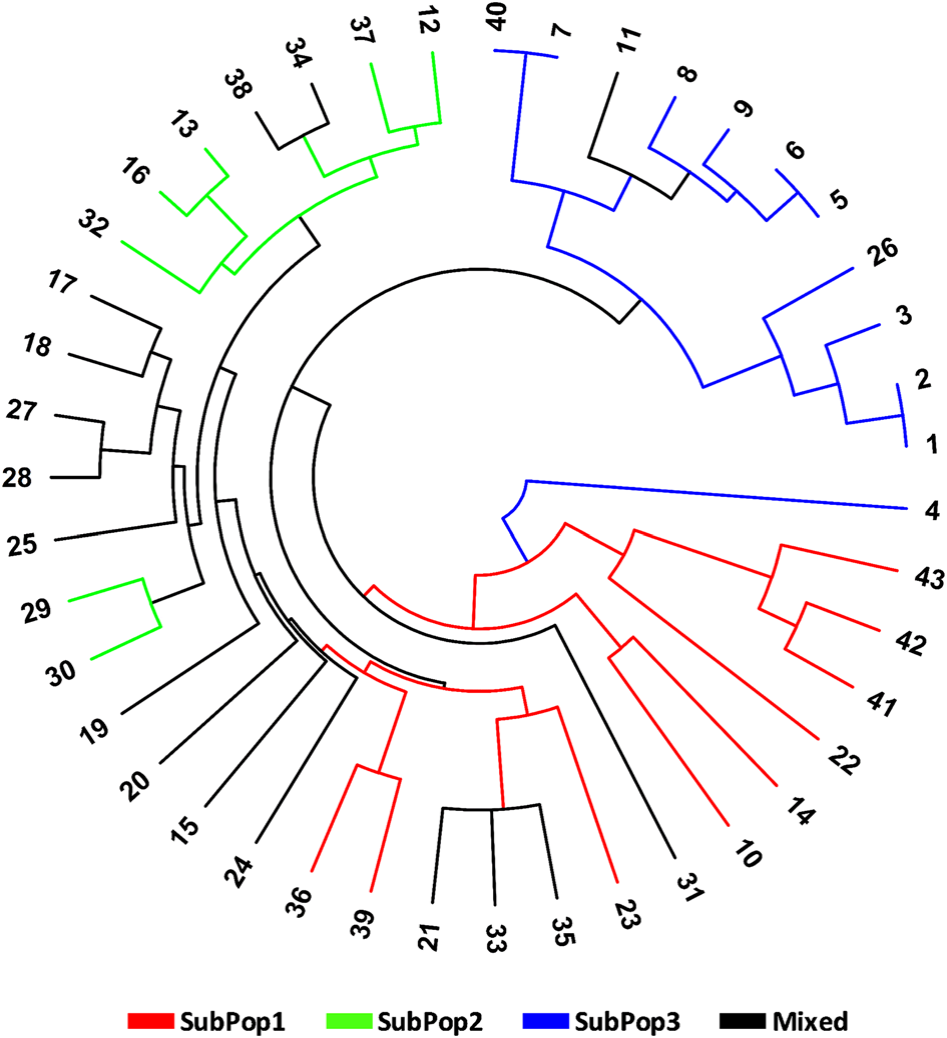
Circular Dendogram of 43 wild barley genotypes constructed from molecular data using OriginPro 2023 software.

Finally, the circular dendrogram generated through multivariate hierarchical cluster analysis using group average method and Jaccard’s coefficient of similarity, also congregated all genotypes into three main clusters analogous to the three subpopulations (Fig. 7) identified in STRUCTURE cluster analysis and PCoA. The dendrogram have been coloured as per the colours revealed by the STRUCTURE software for easy comparison.

**Figure 8:**
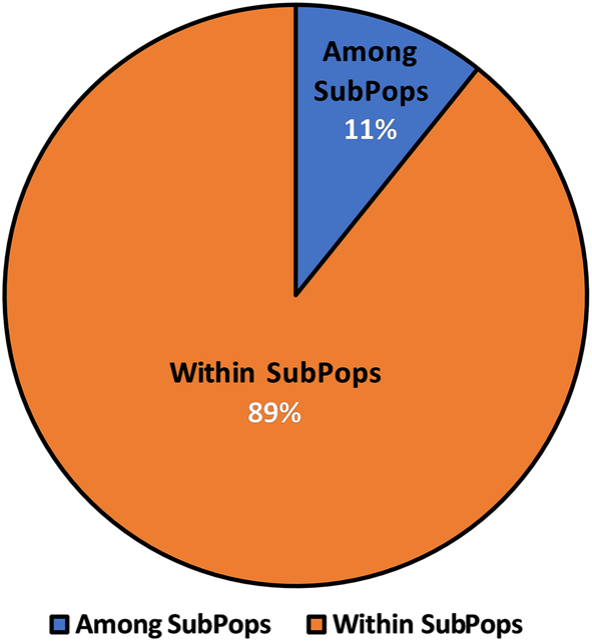
Analysis of molecular variance (AMOVA) of 43 wild barley genotypes based on population obtained by the model-based approach.

Following structural analysis, the three subpopulations were subjected to AMOVA to split genetic variance, investigate structure of the population hierarchically, and identify variation within and across populations. A variance of 11% was reported among subpopulations, while it was 89% within subpopulations (Fig. 8). This suggests substantial gene flow and low intrapopulation differentiation. These subpopulations had interaction with each other to create wide variability among them.

AMOVA produced results that generally concurred with those of structure analysis and the dendrogram based similarity coefficient, confirming the presence of both high genetic diversity and a high population structure level. For doing any further association mapping (AM) study, this is a crucial step.

## Discussion

Understanding and characterization of the population structure and genetic diversity of any crop species is very crucial to plan any genetic improvement programme or conservation strategy. The need for better acclimatizing, high input responsive cultivars and resistance varieties has fuelled the quest for huge ex situ genebank collections^65^. Wild genotypes act as the foundation stone in the process of crop improvement. The formulation of efficient and suitable methods and strategies for utilising the available genetic resources is aided by knowledge of the structure and status of genetic diversity of these wild genotypes. Recently, barley has been seen as a model crop for studies on functional genomics owing to its inbreeding nature and short genome^66^. High genetic diversity is helpful in association mapping, development of genetic marker, building segregating populations and cloning of functional gene^67^. Barley breeding attempts are generally aimed at controlling gene expression by combining and pooling one or more desired traits from donor parental lines to produce superior recombinant offspring^31^.

Different types of molecular or genetic markers have been employed extensively in marker assisted selection (MAS) breeding, population genetic association research, and candidate genes screening. However, it must be noted that each type of DNA marker have unique characteristics that access distinct facets of genetic variation^68^. Microsatellite (SSR) markers are effective tools for discovering genetic correlations between genotypes that would be difficult to recognize physically. Additionally, these markers have been effectively used to identify genetic variation in a variety of crop species, even wild barley^54,69–74^. RFLP markers led to the construction of first barley genetic map^75,76^, however, with progress in molecular biology and biotechnology, PCR based DNA markers like SSR became one of the most useful and informative kind of marker in diversity analysis studies^69^. Based on the ability of microsatellite markers to identify factors like PIC and H, among the various kinds of DNA markers currently employed for barley, have been recognised as the superior markers for MAS breeding programmes, population structure and genetic diversity investigations^77,78^. They are codominant (able to identify heterozygotes), high throughput, multiallelic, highly prevalent and informative markers, apart from the fact that only a tiny amount of DNA sample is needed for analysis, which make its detection in the PCR very easy^50,79^. SSR markers were already being used in research on genetic diversity and population structure, as well as barley breeding since 1994^80–82^. Ramsay et al.^83^ and Li et al.^84^ then combined comprehensive genomic maps with SSR loci. Because there have been few reports of studies using DNA markers to analyse population structure and genetic variability in wild barley genotypes and their response to insect pest infestation, the current study investigated 43 wild barley accessions for genetic diversity and population structure analysis using the 8 microsatellite markers and their association with resistance responses against CLA.

The number of alleles/locus is determined by genotypes, loci, and marker type^55^. The current study used eight polymorphic microsatellite markers on 43 wild barley genotypes to yield 36 alleles ranging from two to seven alleles per locus with a mean of 4.5 alleles/locus. This reveals the existence of beneficial allelic variation, which is regarded to be critical in assessing genetic diversity. This study's findings were consistent with those of Abebe et al.^62^, who found 57 alleles (2–7 range) with an average of 3.8 alleles/marker. Our findings support the findings of several other writers who found alleles per locus ranging from 3.2 to 5.7^31,54,85–88^. The alleles/locus in wild barley (3.12) was greater than in cultivated barley (2.59) when tested using 69 SSR markers, yielding 213 alleles and 3.14 alleles/locus as per the study done by Zhang et al.^59^.

Although the barley genotypes as well as the DNA markers employed in these experiments were different and less compared to other studies, the reported outcomes remained consistent. Some studies have shown striking higher number of alleles per locus such as 7.8 by Maniruzzaman et al.^89^, 8.86 mean number of alleles by Pasam et al.^55^ using a total of 372 alleles from 42 SSR ranging from 3 to 22, 9.4 as reported by Tanto-Hadado et al.^90^ using 7 SSR markers, 9.75 (ranging from 1.25 to 10.67) by Dido et al.^91^, 7.7 by Varshney et al.^92^, 7.6 given by Backes et al.^93^ and 16.7 by Malysheva-Otto et al.^94^. Surprisingly, Elakhdar et al.^42^ observed only 2.6 alleles per locus. Based on different number and forms of alleles, their spread, expressivity and the measurable rate of change in phenotypes of desired genotypes in whole of the population, it is possible to determine the usefulness of the genetic diversity present in those populations^95^. The gene diversity for the studied markers averaged 0.16, ranging from 0.02 to 0.27, which was lower than the average gene diversity (0.41) as reported by Abebe et al.^62^, which ranged from 0.08 to 0.78. This may be due to the less number of markers deployed for the current investigation. Kumar et al.^88^ reported gene diversity of 0.483 and 0.440 for hulled and hulless barley genotypes, respectively. Major allele frequency (MAF) in the present report averaged to 89.28% and scaled from 76.74% to 98.84%. The results of the current report are in harmony with those of Dido et al.^96^ who described that MAF ranged from 0.3 to 0.8. However, the average MAF was much less as reported by Dido et al.^91^ averaging to 0.689 (ranging from 0.531 to 0.784), Pasam et al.^55^ varying from 21.3 to 97.4% with an average of 51.2% and Kumar et al.^88^ ranging from 0.271 to 0.917 with a mean of 0.532.

Private alleles are used in conservation genetics to quantify the amount of gene flow across populations^97,98^. Half of the alleles were unique/private alleles found in 19 wild barley genotypes (44.18% of total genotypes) in the current investigation. The abundance of private/unique alleles indicates that these genotypes may be used in barley breeding programmes as sources of novel genes. Consequently, the data from this study will make a substantial contribution to strategies for improving barley genotypes. In contrast with the findings of the present report, Tanto-Hadado et al.^90^ and Dido et al.^91^ reported to have 23% (in 15 barley genotypes) and 19.25% (in 92 barley landraces) of the total allels as private alleles, respectively. SSR markers' capacity to differentiate between genotypes is known as their PIC, and it is largely dependent on allelic diversity. According to Botstein et al.^99^, the PIC index may be used to measure the degree of gene variation. The eight markers utilised in the current research had PIC values ranging from 0.35 (MS28) to 0.80 (MS31), averaging to 0.66, showing that they were highly informative. Among the markers used, 87.5% had PIC > 0.5 and hence, regarded as extremely informative and polymorphic markers. However, Abebe et al.^62^ observed only 47% and 33% of the SSR markers as highly and slightly informative. Several studies including those by Macaulay et al.^100^, Malysheva-Otto et al.^94^, Jilal et al.^101^, Naeem et al.^52^, Pasam et al.^55^, Hua et al.^102^, Dido et al.^96^ and Dido et al.^91^ had reported comparable average PIC values of 0.64, 0.69, 0.78, 0.58, 0.548 (ranging from 0.050 to 0.839 of 42 SSR markers of which 78% had 0.4 < PIC < 0.8), 0.549 (ranging from 0.064 to 0.815), 0.552 and 0.727 (ranging from 0.243 to 0.883 of 49 microsatellite markers of which 32 (65.3%) had PIC > 0.727), respectively to the PIC values of the current study. However, there had been few studies which reported PIC values of ≤ 0.5 for the SSR markers used on barley genotypes^31,42,86,88,103^. Furthermore, PIC values in a research conducted by Zhang et al.^59^ ranged between 0.08 and 0.75 (0.46 mean), and the PIC of cultivated barley (0.37) was less than wild barley (0.44). In 43 genotypes of *H. vulgare* subsp*. spontaneum*, the microsatellite markers utilised in this research produced repeatable polymorphic bands, demonstrating their effectiveness as a molecular marker for examining genetic diversity, population structure, and interactions among wild barley genotypes. The SSR markers show considerable genetic diversity among and within these wild barley genotypes, despite the modest number of genotypes and markers utilised in the present investigation.

The presence of high genetic variability among the genotypes and subpopulations was confirmed by the broad range of GS varying from 0.55 to 1.00 and averaging to 0.82. The vast and rich diversity found using highly polymorphic markers is evident in the broad similarities across and within the subpopulation in the investigated population. Pandey et al.^86^ reported presence of genetic similarity averaging 0.50 (ranging from 0.24 to 1.00), while, in another study conducted by Elakhdar et al.^31^ reported genetic similarity of upto 0.80 among Egyptian barley genotypes. The top three principal components (PC) contributed 44.51% of the overall variance, according to principal coordinate analysis (PCoA), of which PC1, PC2 and PC3 make up for 17.15%, 16.28% and 11.08% of the variance, respectively. The total variation observed in the current study is by far more than those observed by Abebe et al.^62^, who reported a total variation of 28.43% with the three components contributing 11.80%, 8.72% and 7.91%, respectively. Moreover, the set of markers and barley genotypes used by Malysheva-Otto et al.^94^ and Kumar et al.^88^ in their PCoA analysis using the first two components could only explain 14.8% (8.9% and 5.9%) and 20% of the total variation, respectively. In order to segregate genetic variance and inspect at hierarchical population structure, analysis of molecular variance (AMOVA) was also carried out. The genetic diversity was mostly derived from variance within accessions (89%) than among genotypes (11%). The genetic diversity seen within each accession, suggests that even an individual accession might serve as a source of breeding material^54,104^. These results accords with more than 80% variation within clusters described by Dido et al.^96^ and Dido et al.^91^ in the Ethiopian barley genotypes. On the other hand, Elakhdar et al.^31^ and Abebe et al.^62^ reported same trend of variation in barley genotypes but with high molecular variation among genotypes of 49% and 27.77%, respectively. Contrarily, Kumar et al.^88^ observed more variation among genotypes (78%) in comparison to within genotypes (22%). The diversity of the barley genotypes included in the study showed that a large proportion of variation could be explored with a small number of representative samples, suggesting that focusing on individual genotypes during sampling is more valuable, time saving and cost effective, compared to focusing on regions.

The current study is unique as it studied the genetic diversity and population structure level of 43 wild barley genotypes for resistance to the corn-leaf aphid, *R. maidis* first time in India in order to set the groundwork for future aphid resistance breeding programmes. The 3 statistical approaches viz*.* STRUCTURE analysis, PCoA and circular dendrogram gave uniform results for the genetic diversity and population structure analysis. Based on these various clustering analysis, all the 43 wild barley genotypes were divided into three subpopulations (K = 3). Based on 66.66% Q, the high number of admixture genotypes (37.20%) in the present study, suggests that these genotypes are extremely diversified in spite of being sampled from the same geographical location. Among all subpopulations, the genotypes belonging to SubPop3 (Blue) with the lowest mean aphid infestation (8.78 aphids/shoot) and the maximum number of pure genotypes (i.e., 11) emerged as the most promising group for the search for aphid resistance. Previous authors have exploited the genetic diversity in various barley genotypes for searching sources of resistance against various fungal diesases^33,96,105–108^, viral diseases^109,110^ and insect pests like barley shoot fly^32^. But none had studied the genetic diversity and population structure analysis for resistance against *R. maidis* in wild barley germplasm. The studies conducted by several authors like Tanto-Hadado et al.^90^, Pasam et al.^55^ Muñoz-Amatriaín et al.^41^, Elakhdar et al.^42^, Elakhdar et al.^31^, Kumar et al.^88^, Dido et al.^96^ and Abebe et al.^62^ completely justifies the findings of the present investigation with K = 2 (Q ≥ 80%), K = 4, K = 2 (Q ≥ 70%), K = 5, K = 3, K = 2, K = 3 and K = 2 (Q ≥ 80%) in their respective studies. However, some studies reported higher number of clusters in the barley populations. Pandey et al.^86^ and Zhang et al.^59^ reported eight subpopulations in the barley genotypes with 20% (based on Q ≥ 70%) and 36.5% admixtures, respectively. Dido et al.^91^ reported that 49 SSR markers used in his research separated 384 individual genotypes of Ethiopian barley landraces into seven clusters (K = 7) with 48 admixture genotypes (12.5%). The clustering pattern could not be compared due to the diverse set of genotypes and markers used in each of the studies mentioned above.

Based on the AII, six (14%), six (14%), 19 (44%) and 12 (28%) of the 43 wild barley genotypes were characterized as resistance, moderately resistance, susceptible and highly susceptible genotypes to *R. maidis*, respectively. There was no immune genotype identified in the current investigation. The findings of this study are in line with that of Hsu and Robinson^111,112^, Chhillar et al.^113^, Singh et al.^114^, Kumar et al.^115^ and Kumar et al.^116^, who reported that that none of the barley varieties tested against *R. maidis* were found to be immune to the pest. In India, there are few sources of resistance to corn leaf aphid^117^, and no cultivars have been introduced for this trait. However, recently two resistant genotypes BCLA3 and BCLA11-6 have been registered as genetic stocks for novel source of corn leaf aphid resistance at NBPGR, New Delhi^118^. It was observed that genotypes employed in the previous investigations were largely cultivated barley and not wild barley genotypes, therefore findings of this study are novel and can useful for breeders who are developing aphid resistance barley cultivars.

Since, almost all the subpopulations had genotypes from all places of origin of genotypes used in the current investigation, there was no association between the clustering pattern and the region of collection of genotypes. Abebe et al.^62^ reported the assertion that the genetic variations recorded in the barley landraces are not because of geographic locations but due to highly diversified genotypes collected from the same region resulting in higher proportion of admixtures. Different researchers suggested a lack of robust and distinct demographic clustering patterns based on the geographic location of barely landraces^91,119–122^. Dido et al.^96^ in his study also confirmed the absence of geographical structure. Orabi et al.^123^ suggested that the place of origin didn’t impact the grouping, except for the genotypes from Morocco in his study on diversity analysis of cultivated and wild barley from North Africa and West Asia. Surprisingly, Pasam et al.^55^ found good fit of accessions into geographical clusters. The results of all of the aforementioned approaches were found to be similar with each other solidifying the notion of presence of both large genetic variability and a high level of population structure. The observed genetic diversity within and between accessions supported the possibility of using these genotypes as a source of barley aphid resistance breeding materials. Pure lines generated from genotype combinations can be utilised as a parental line, an improved cultivar, or in mixtures to increase yield stability, insect pest resistance, and disease tolerance. The variability present within a crop species affects genetic improvement of yield and contributing characteristics, and the barley genotypes with high levels of genetic variation found in this study are valuable resources for extending the genetic base and achieving rapid improvements in barley breeding programmes around the world.

## Conclusions

In conclusion, for any breeding programme targeting resistance, genotype selection should be done carefully. Cluster analysis categorized all the 43 wild barley genotypes into 3 subpopulations. Significant (*p* < 0.05) differences were found among subpopulations, which indicates diversification among barley genotypes. The cluster analysis results indicated a large degree of genetic diversity and population structure, indicating that they can be used in resistance breeding program. As a result, the germplasm and highly polymorphic microsatellite markers discovered during this work have the potential to help build *R. maidis* resistance breeding schemes. This study's findings provide a solid foundation for future research on the mechanism of corn-leaf aphid resistance in barley.

## Methods

### Genotype screening

The lab and screening experiments were performed at the Entomology Laboratory and Research Field of the ICAR-Indian Institute of Wheat and Barley Research (IIWBR), Karnal (India) during *rabi* season 2021–22. A set of 43 wild barley (*H. vulgare* subsp*. spontaneum*) genotypes were acquired from the IIWBR GRU (Germplasm Resources Unit) facility and screened for *R. maidis* resistance response. The plant materials utilized in the present study conform to relevant international, national and institutional guidelines. Supplementary Table S1 online lists all the wild barley genotypes with their origins which have been screened during the present investigation. Each genotype was sown in a meter row length with 25 cm row to row spacing with 3 replications (2 rows per replication) in RBD (randomized block design) following all barley package and practices except any insecticide treatment. Each genotype was allotted genotype code and name (accession number) and tagged accordingly. Five randomly chosen plants from each genotype row were screened three times during the season for aphid resistance by counting number of aphids per shoot. The classification of wild barley genotypes was done on the basis of grading system proposed by Zhu et al.^124^ as mentioned in the Supplementary Table S2 online. Based on these scores, an Aphid Infestation Index (AII) was devised according to Kumar et al*.*^115^ with slight modifications. For calculation of AII, aphids per shoot, leaf chlorosis and leaf rolling were weighted in 3:2:1 ratio, respectively. The index varied from 0 to 5 on ordinal scale with 0 indicating immune and 5 indicating highly susceptible genotype.

### Plant material sampling and DNA extraction

Each of the 43 wild barley genotypes’ four to five tender leaf samples from 30 days old barley seedlings were cut using scissors, collected in aluminium packets, and taken in an ice box to the Entomology Laboratory. DNA was extracted from these collected leaf samples following a 3-day long Cetyl Trimethyl Ammonium Bromide (CTAB) extraction method^125^ with minor modifications. To remove the RNA impurities from the sample, 3 µl of ready-to-use RNase (10 mg/ml) was added to each DNA sample. The quality (A_260_/A_280_ absorbance ratio: stock DNA purity) and quantity (concentration of stock DNA) in ng/μl was determined by loading 1 µl sample of stock DNA of each genotype in the ScanDrop Spectrophotometer (Analytik Jena). On the basis of these parameters, the stock DNA concentration was reduced to 50 ng/μl by adding NFW (Nuclease Free Water) to prepare working solution of each genotype.

In the current investigation, 15 SSR primers from barley genome were selected by reviewing the literature. After preliminary primer run, eight polymorphic SSR primers (Table 2) specific to aphid were shortlisted by eliminating the seven monomorphic primers for screening of barley genotypes against *R. maidis* infestation. The working primer solution was prepared by diluting 10 μl stock primer solution (100 pmol/μl) with 90 μl NFW (1:9 ratio). The details of the primers have been listed in Table 2, which were synthesized by Eurofins Genomics India Private Limited, Bangalore.

### Polymerase chain reaction (PCR)

A 10 µl PCR reaction was carried out constituting GoTaq^®^ Green Master Mix (5 µl), forward primer (0.3 µl), reverse primer (0.3 µl), NFW (3.4 µl) and 1 µl DNA of 50 ng/μl concentration. To execute amplification reactions, a 96 gradient Q-Cycler 96 (Hain Lifescience, UK) was used. The thermal cycling parameters used to run the PCR included initial denaturation at 94 °C (4 min), followed by 35 cycles of denaturation at 94 °C (60 s), annealing at 52 °C (MS25 and MS27) and 54 °C (MS3, MS17, MS20, MS21, MS28, and MS31) for 60 s, extension at 72 °C (1 min), and final extension at 72 °C (7 min). The amplification products of PCR were resolved on standard agarose gel electrophoresis (3%) mixed with ethidium bromide fluorescent dye at 90 V (1.5 h) using 100 bp Promega DNA ladder (5 μl) for amplicon size comparision. This was followed by visualization and image capture under UV gel documentation unit (Vilber). For each SSR primer, amplicons of the uniform size present in different genotypes were considered as same allele.

### Analysis of population structure and phylogenetic relationships

For further analysis, the bands were rated as one (“1” = presence of band) or zero (“0” = absence of band), resulting in a raw data matrix that could be utilised as input in several genetic analysis softwares. The genotypic data of the number of alleles per marker and the base pair size of the alleles were used to compute the Heterozygosity (H) and Polymorphic Information Content (PIC) of each microsatellite marker using Gene-Calc^126^. DNA markers with a PIC more than 0.5 are considered highly informative and polymorphic markers^99^, whereas 0.5 > PIC  > 0.25 and PIC lesser than 0.25 are considered medium and low informative markers, respectively ^127^. The UPGMA (unweighted pair group method with arithmetic averages) coefficient of Jaccard’s similarity-based clustering method was used to construct a genetic similarity (GS) matrix in NTSYS-pc 2.0 (Numerical Taxonomy and Multivariate Analysis System for Personal Computer by Exeter software, USA) software^128^. PowerMarker 3.25 was used to provide summary statistics data on the number of alleles, gene diversity, and the frequency of major alleles (MAF)^129^. Three different statistical approaches were applied and compared to examine the population structure of the 43 wild barley genotypes. Initially, a Bayesian model-based clustering technique with STRUCTURE 2.3.4 software admixture model with correlated frequency of alleles was used to determine the precise number of subpopulations (value of K)^130,131^. To get an accurate estimate of K, three replications were computed for each assumed value of K (or the number of subpopulations) ranging from 1 to 12, with a 100,000 iteration burn-in phase followed by a hundred thousand Markov Chain Monte Carlo iterations (MCMC Reps). The STRUCTURE harvester was used to collect analysis output^132^. The number of most obvious and expected subpopulations (indicated by the best/highest value of K) was estimated using an ad hoc statistic using Evanno’s K^63^ and the LnP(D) (data log probability) based on the differential of LnP(D) between successive K values^132^. CLUMPP was used to plot the graph of structure of population to find the mean of each run^133^. All the genotypes were allocated to their respective subpopulations on the basis of coefficient of membership probability (Q) ≥ 0.6666 (66.66%) which means that any genotype with two-third of its membership coefficient belonging to any of the subpopulation was classified as the pure genotype of that subpopulation. The admixtures were grouped in a separate group of “Mixed” subpopulation, however, they were assigned as admixture genotype of a subpopulation with highest proportion of Q. All these genotypes were sorted by Q which represents the probability of an individual belonging, partially or fully to one or more subpopulations under investigation and clubs them together in ordinal manner. Secondly, PCoA (Principal coordinate analysis) using PAST software 4.08 was performed by reducing the data dimensions into mainly two principal components to characterize the genetic stratum of wild barley germplasm based on correlations among the subpopulations and their genotypes^134^. Finally, using OriginPro^®^ 2023^135^ (OriginLab Corporation, USA) software and multivariate hierarchical cluster analysis based on marker data, the phylogenetic connection among the 43 wild barley genotypes was assessed. The genetic distance matrix was used to construct the coefficient of Jaccard’s similarity-based circular dendrogram (phylogenetic tree). The major trends of variation in the multilocus dataset were summarised using an analysis of molecular variance (AMOVA) utilising Excel addins software GenAlEx (Genetic Analysis in Excel) 6.502^136^.

### Statistical analysis

The phenotypic data of mean number of aphids infesting the 43 wild barley genotypes was superimposed on the categorization as revealed by the STRUCTURE cluster analysis. The variability between subpopulations and among whole of the population was compared using boxplot showing descriptive statistics parameters like interquartile range, size of subpopulation, median and range in SPSS software (SPSS Statistics 25.0, IBM)^137^. To compare the means of number of aphids infesting genotypes of various subpopulations, One-way ANOVA (analysis of variance) (SPSS Statistics 25.0, IBM)^137^ was carried out at *p* < 0.05 (5% significance level). Further, post-hoc comparisons were done using Dunnett’s T3 test to check differences between different subpopulation pairs. All the genotypes were categorized into different resistance responses based on Aphid Infestation Index (AII) using a pie-chart (Excel 2019, Microsoft Company)^138^. At last, the data of mean number of aphids per shoot, the subpopulation categorization by STRUCTURE and the AII based resistance responses was integrated in a Wind-Rose Chart (Excel 2019, Microsoft Company)^138^ for identification of the desired aphid resistant genotypes and breeding material.

### Supplementary Information

Supplementary Tables.

## Data Availability

The raw data supporting the conclusions of this article will be made available by the corresponding author, without undue reservation, to any qualified researchers.
