# Supplementary material for: Deciphering the genetic diversity and population structure of wild barley germplasm against corn leaf aphid, Rhopalosiphum maidis (Fitch)
Source: Sci Rep. 2023 Oct 12;13:17313. doi: 10.1038/s41598-023-42717-7 (PMC10570286; doi:10.1038/s41598-023-42717-7)
Supplement: Supplementary file 1 — Supplementary Tables. [file 41598_2023_42717_MOESM1_ESM.doc]

**Deciphering the genetic diversity and population structure of wild barley germplasm**

**against corn leaf aphid, *Rhopalosiphum maidis* (Fitch)**

Sunny Maanju^1,2^, Poonam Jasrotia^1^*, Surender Singh Yadav^2^, Prem Lal Kashyap^1^, Sudheer Kumar^1^, Manoj Kumar Jat^2^, Chuni Lal^1^ , Preeti Sharma^2^, Gyanendra Singh^1^ & Gyanendra Pratap Singh^1,3^

*^1^ICAR- Indian Institute of Wheat and Barley Research, Karnal 132001, Haryana, India*

*^2^CCS Haryana Agricultural University, Hisar 125004, Haryana, India*

*^3^ICAR- National Bureau of Plant Genetic Resources, New Delhi 110012, India*

**Corresponding author:**

*Poonam Jasrotia, Principal Scientist, ICAR-Indian Institute of Wheat and Barley Research, Karnal 132001, Haryana, India.

Tel: (+91) 184-2209197

E-mail: poonam.jasrotia@icar.gov.in, [poonamjasrotia@gmail.com](mailto:poonamjasrotia@gmail.com)

| **Genotype code** | **Genotype name/ Accession number** | **Acquisition year** | **Provenance/ Origin** | **Site code** | **PDCI score (out of 10)** |
| --- | --- | --- | --- | --- | --- |
|  | IG 38780 | 1987 | Israel | Unknown | 7.5 |
|  | IG 38931 | 1987 | Israel | PAL-S59 | 8.9 |
|  | IG 39127 | 1987 | Israel | PAL-S138 | 8.3 |
|  | IG 39565 | 1987 | Palestinian Territory | PAL-S151 | 8.9 |
|  | IG 135854 | 2002 | Jordan | JOR02-2:24 | 9.6 |
|  | IG 142356 | 2006 | Tajikistan | TJK06:5 | 9.6 |
|  | IG 142486 | 2008 | Tajikistan | TJK06:29 | 9.0 |
|  | IG 144112 | 2008 | Jordan | JOR81-2:7581 | 9.0 |
|  | IG 144113 | 2008 | Syria | SYR80-2:510 | 9.0 |
|  | IG 144114 | 2008 | Syria | SYR80-2:564 | 9.0 |
|  | IG 144116 | 2008 | Israel | PAL-S48 | 8.7 |
|  | IG 144117 | 2008 | Israel | PAL-S80 | 8.1 |
|  | IG 144121 | 2008 | Syria | SYR88-2:10 | 9.9 |
|  | IG 144123 | 2008 | Jordan | JOR91:42 | 9.9 |
|  | IG 144124 | 2008 | Jordan | JOR91:44 | 9.9 |
|  | IG 144127 | 2008 | Jordan | JOR91:72 | 9.9 |
|  | IG 144128 | 2008 | Jordan | JOR91:85 | 9.9 |
|  | IG 144129 | 2008 | Syria | SYR92-3:9 | 9.9 |
|  | IG 144157 | 2008 | Jordan | JOR08:1 | 9.9 |
|  | IG 144161 | 2008 | Jordan | JOR08:4 | 9.9 |
|  | IG 144898 | 2009 | Libya | LBY09:1 | 9.9 |
|  | IG 144903 | 2009 | Libya | LBY09:3 | 9.9 |
|  | IG 144911 | 2009 | Libya | LBY09:8 | 9.9 |
|  | IG 144913 | 2009 | Libya | LBY09:9 | 9.9 |
|  | IG 144927 | 2009 | Libya | LBY09:16 | 9.9 |
|  | IG 144930 | 2009 | Libya | LBY09:18 | 9.9 |
|  | IG 144933 | 2009 | Libya | LBY09:20 | 9.9 |
|  | IG 144951 | 2009 | Libya | LBY09:31 | 9.9 |
|  | IG 144983 | 2009 | Libya | LBY09:49 | 9.9 |
|  | IG 145080 | 2009 | Syria | SYR09-1:1 | 9.9 |
|  | IG 145494 | 2009 | Tajikistan | TJK09:1 | 5.85 |
|  | IG 145498 | 2009 | Tajikistan | TJK09:2 | 5.85 |
|  | IG 145502 | 2009 | Tajikistan | TJK09:3 | 5.85 |
|  | IG 145504 | 2009 | Tajikistan | TJK09:4 | 5.85 |
|  | IG 145508 | 2009 | Tajikistan | TJK09:5 | 5.85 |
|  | IG 145523 | 2009 | Tajikistan | TJK09:9 | 5.85 |
|  | IG 145528 | 2009 | Tajikistan | TJK09:11 | 5.85 |
|  | IG 145539 | 2009 | Tajikistan | TJK09:13 | 5.85 |
|  | IG 145556 | 2009 | Tajikistan | TJK09:19 | 5.85 |
|  | IG 145597 | 2009 | Tajikistan | TJK09:33 | 5.85 |
|  | IG 145602 | 2009 | Tajikistan | TJK09:35 | 5.85 |
|  | IG 145604 | 2009 | Tajikistan | TJK09:36 | 5.8 |
|  | IG 145610 | 2009 | Tajikistan | TJK09:39 | 5.85 |

**Table S1.** List of wild barley (*Hordeum vulgare subsp. spontaneum*) genotypes under investigation. Source: Genesys (<https://www.genesys-pgr.org/>); PDCI: Passport Data Completeness Index.

| **Grading based on aphid population (Scale 1 to 5)** | | |
| --- | --- | --- |
| **Grade/Scale** | **Number of aphids per shoot** | **Rating/Reaction** |
| 1 | 0 | Immune (I) |
| 2 | 1 to 5 | Resistant (R) |
| 3 | 6 to 10 | Moderately Resistant (MR) |
| 4 | 11 to 20 | Susceptible (S) |
| 5 | 21 and above | Highly Susceptible (HS) |
| **Grading based on leaf chlorosis (Scale 1 to 5)** | | |
| **Grade/Scale** | **Leaf chlorosis** | **Rating/Reaction** |
| 1 | No chlorosis | Immune (I) |
| 2 | Less than 1/3 of leaf area chlorotic | Resistant (R) |
| 3 | 1/3 to 2/3 leaf area chlorotic | Moderately Resistant (MR) |
| 4 | More than 2/3 of leaf area chlorotic | Susceptible (S) |
| 5 | Necrosis in at least one full leaf | Highly Susceptible (HS) |
| **Grading based on leaf rolling (Scale 1 to 3)** | | |
| **Grade/Scale** | **Leaf rolling** | **Rating/Reaction** |
| 1 | No rolling | Resistant (R) |
| 2 | Trapping or curling in one or more leaves | Moderately Resistant (MR) |
| 3 | Rolling in one or more leaves | Susceptible (S) |

**Table S2:** Grading system followed for wild barley genotypes screening against *R. maidis*

| **Grade** | **Reaction** | **No. of Genotypes** | **Wild Barley Genotypes** |
| --- | --- | --- | --- |
| **1** | **Immune (I)** | **0** | -Nil- |
| **2** | **Resistant (R)** | **6** | IG 135854, IG 142356, IG 142486, IG 144112, IG 144113 and IG 144114 |
| **3** | **Moderately Resistant (MR)** | **6** | IG 38780, IG 38931, IG 39127, IG 39565, IG 144930 and IG 145597 |
| **4** | **Susceptible (S)** | **19** | IG 144117, IG 144121, IG 144123, IG 144127, IG 144129, IG 144157, IG 144161, IG 144898, IG 144911, IG 144927, IG 144933, IG 144951, IG 144983, IG 145080, IG 145498, IG 145502, IG 145504, IG 145523 and IG 145556 |
| **5** | **Highly Susceptible (HS)** | **12** | IG 144116, IG 144124, IG 144128, IG 144903, IG 144913, IG 145494, IG 145508, IG 145528, IG 145539, IG 145602, IG 145604 and IG 145610 |

**Table S3:** Characterization of wild barley genotypes into various categories of plant resistance based on AII.
